# Community-based cross-sectional survey of latent tuberculosis infection in Afar pastoralists, Ethiopia, using QuantiFERON-TB Gold In-Tube and tuberculin skin test

**DOI:** 10.1186/1471-2334-11-89

**Published:** 2011-04-09

**Authors:** Mengistu Legesse, Gobena Ameni, Gezahegne Mamo, Girmay Medhin, Gunnar Bjune, Fekadu Abebe

**Affiliations:** 1 Aklilu Lemma Institute of Pathobiology, Addis Ababa University, Addis Ababa, Ethiopia; 2 Faculty of Veterinary Medicine, Addis Ababa University, Beshofitu, Ethiopia; 3 Department of General Practice and Community Medicine, Institute for Health and Society, University of Oslo, Oslo, Norway

## Abstract

**Background:**

There is little information concerning community-based prevalence of latent tuberculosis infection (LTBI) using T-cell based interferon-γ (IFN-γ) release assays (IGRAs), particularly in TB endemic settings. In this study, the prevalence of LTBI in the Afar pastoral community was assessed using QuantiFERON-TB Gold In-Tube (QFTGIT) and tuberculin skin tests (TST).

**Methods:**

A community-based cross-sectional survey of LTBI involving 652 apparently healthy adult pastoralists was undertaken in the pastoral community of Amibara District of the Afar Region between April and June 2010.

**Results:**

The prevalence of LTBI was estimated as 63.7% (363/570) using QFTGIT at the cut-off point recommended by the manufacturer (≥ 0.35 IU/ml IFN-γ), while it was 74.9% (427/570) using a cut-off point ≥ 0.1 IU/ml IFN-γ. The QFTGIT-based prevalence of LTBI was not significantly associated with the gender or age of the study participants. However, the prevalence of LTBI was 31.2% (183/587) using TST at a cut-off point ≥ 10 mm of skin indurations, and it was higher in males than females (36.8% vs. 23.5%, X^2 ^= 11.76; p < 0.001). There was poor agreement between the results of the tests (k = 0.098, 95% CI, 0.08 - 0.13). However, there was a positive trend between QFTGIT and TST positivity (X^2 ^= 96.76, P < 0.001). Furthermore, individuals with skin indurations ≥ 10 mm were 13.6 times more likely to have positive results using QFTGIT than individuals with skin indurations of 0 mm (adjusted OR = 13.6; 95%CI, 7.5 to 24.7, p < 0.001).

**Conclusions:**

There is currently no agreed gold standard for diagnosis of LTBI. However, the higher prevalence of LTBI detected using QFTGIT rather than TST suggests that QFTGIT could be used for epidemiological studies concerning LTBI at the community level, even in a population unreactive to TST. Further studies of adults and children will be required to assess the effects of factors such as malnutrition, non-tuberculosis mycobacterial infections, HIV and parasitic infections on the performance of QFTGIT.

## Background

Tuberculosis (TB) is one of the major public health problems in sub-Saharan Africa and Asia [1]. Globally, it is responsible for approximately two million deaths, and eight million new cases are reported each year; approximately 80% of all new cases occur in the 22 countries with a high burden of TB [2]. Furthermore, it is estimated that one third of the world's population has LTBI [3]. This global prevalence of LTBI was estimated predominantly on the basis of data obtained from the tuberculin skin test (TST) survey. However, TST has several limitations including a high rate of false-positive results among individuals vaccinated with bacille Calmette-Guérin (BCG) or exposed to non-tuberculosis mycobacteria [4,5], and a high rate of false-negative results among immune-suppressed individuals [6]. Therefore, uncertainty remains concerning the accuracy and reliability of this previously estimated global prevalence of LTBI [7].

Recently, T-cell-based IFN-γ release assays (IGRAs) have been developed and approved for the diagnosis of LTBI [8], and one study demonstrated that IGRAs have a higher specificity than TST as the results are not affected by the BCG status of the subject [9]. In addition, these tests are affected less by anergy than TST [10], although their sensitivities are questioned by some studies [11,12]. IGRAs have limitations including the inability to differentiate between active TB and past infection [13], and concerns regarding the validity of the present cut-off values recommended by the manufacturers [14]. However, regardless of their limitations, it is believed that IGRAs could improve existing information about the global epidemiology of LTBI [15], but the majority of studies concerning LTBI and IGRAs have been limited to patients with active TB or health care workers and refugees [16-20]. Few studies have assessed community-based prevalence of LTBI using IGRAs [21,22 ].

Ethiopia is ranked 7^th ^among the 22 countries with a high-burden of TB and second in Africa [1]. It is expected that there are a large number of reservoirs of LTBI in Ethiopia, although there are no reliable data and available information is based on the results of TST surveys conducted several years ago [23,24]. Afar Region is one of the main pastoral areas of Ethiopia, where TB is a major public health problem [1,25], although the extent of LTBI is unknown. In this study, the prevalence of LTBI in the Afar pastoral community was assessed using QFTGIT and TST.

## Methods

### Study area and population

A community-based cross-sectional study was conducted in the Amibara District of the Afar Region, north-east Ethiopia, between April and June 2010. Amibara District is located in the Middle Awash valley ~260 km to the north-east of Addis Ababa, the capital city of Ethiopia. The study area has been described in detail elsewhere [26].

### Sample size determination and data collection

There was no previous information concerning the general prevalence of LTBI in the Afar Region, or in the Amibara District specifically. From the assumption that 50% of the study participants would have LTBI, and the following targets: a 95% confidence level, a 5% degree of accuracy, a design effect of 1.5, 10% compensation for non-respondents, 10% compensation for exclusion during screening and 15% compensation for those participants lost to follow up; it was hoped to enrol a total of 713 study participants. Individuals were considered eligible for participation if they were apparently healthy, aged over 18 years, not pregnant (females), able to provide a three ml blood sample, volunteered to be injected with purified protein derivative (PPD), and gave written consent.

Prior to data collection, a list of all the pastoral *kebeles *(smallest administrative units) in the district was obtained from the District Health Office. On the basis of this list, nine pastoral kebeles were randomly selected out of a possible 14 kebeles. The selected kebeles were stratified into manageable villages (if there was more than one village in the kebele) and a list of the head of households of each kebele/village was noted. Using the number of households in each kebele, the pre-estimated sample size (713) was proportionally distributed between these kebeles. The required number of households from each kebele/village was selected using systematic random sampling from the lists of the heads of the households of that kebele/village. From each of the selected households, one individual (male or female) was systematically selected and requested to participate in the study. In the event of refusal, the next household on the list was asked to participate. Participants were requested to attend a respective health post or central place of each Kebele for clinical examination and a skin test and to provide a blood sample. Each study participant underwent a clinical and physical examination and was interviewed to ascertain any previous history of TB or contact with TB patients, and to investigate the presence of other acute or chronic illness, using a questionnaire. Information concerning socio-demographic characteristics of the participants was included in the questionnaire.

### QuantiFERON-TB Gold In-Tube assay

The QFTGIT assay was performed according to the manufacturer's instructions (QuantiFERON-TB Gold In-Tube, Cellestis Ltd., Carnegie, Australia). Briefly, a venous blood sample (1 ml) was collected from each individual into three tubes (one containing TB-specific antigens, one containing mitogen and a nil tube). The samples were transported to Awash Health Centre within 4-6 h of collection and incubated for 24 h at 37°C. The samples were centrifuged at 3000 × rcf (relative centrifugal force) for 10 min, and the plasma was collected and stored at 4°C until the IFN-γ assay was performed using ELISA kits provided with the TB-Gold tube. The optical density (OD) of each test was read using a 450 nm filter with a 620 nm reference filter, using an ELISA plate reader. The results were interpreted as positive, negative or indeterminate on the basis of the manufacturer's recommended cut-off value (IFN-γ ≥ 0.35 IU/ml) using QFTGIT analysis software developed by the company.

Prior to its utilization for the present epidemiological study, the performance of QFTGIT in the Afar Region was evaluated in patients clinically suspected of having active pulmonary TB (PTB) and in healthy control subjects using a different study design and different study participants [27]. The QFTGIT results were interpreted using the population-specific cut-off point (IFN-γ ≥ 0.1 IU/ml), which was estimated by receiver operator characteristic (ROC) curve analysis [27] prior to the implementation of the study.

### Tuberculin skin test

Immediately following blood collection from the right hand, 0.1 ml (2T.U/0.1 ml) tuberculin PPD RT23 (Statens Serum Institute, Copenhagen, Denmark) was administrated intradermally in the middle of the left forearm by an experienced nurse. The diameter of the indurations was measured transversely after 48-72 h using a ball-point pen and flexible plastic ruler [28]. Diameters of skin indurations ≥ 10 mm were considered positive, while diameters of 0 mm were considered non-responsive [29,30].

### Ethical considerations

The study protocol was approved by the Ethical Clearance Committee of the Aklilu Lemma Institute of Pathobiology (ALIPB), Addis Ababa University, and by the Regional Committee for Medical Research Ethics of Southern Norway. The aim of the study was explained to each of the study participants and written consent was obtained. Blood sample collection and tuberculin PPD injections were carried out under aseptic conditions by well experienced technicians and nurses, respectively. Individuals who presented with signs/symptoms of active TB or other diseases were advised to attend the nearest health centre. Similarly, individuals whose TST indurations were >10 mm and/or tested positive for LTBI using QFTGIT were advised to consult the nearest health facilities lest they develop signs/symptoms suggestive of active TB.

### Data analysis

Data were entered into EpiData Software v.3.1 and analyzed using Stata version 11. Frequencies and percentages were used to summarise socio-demographic characteristics and the prevalence of LTBI as diagnosed using QFTGIT and TST. The prevalence of LTBI was estimated by dividing the number of participants with positive TST or QFTGIT results by the total number of study participants who had undergone the TST or QFTGIT test. The combined prevalence of LTBI was estimated by dividing the number of participants who tested positive using TST and QFTGIT by the total number of study participants who had test results for both TST and QFTGIT. Pearson's chi-square was used to compare the proportions of target outcomes. Univariable logistic regression analysis was performed to assess the association between LTBI and background characteristics of study participants including age, sex, and history of BCG or close contact with TB patients. Multivariable logistic regression analysis was used to assess the effect of each of the independent variables (such as gender, age, occupation and marital status) on the outcome variable by adjusting each independent variable for all other variables. A p-value less than 0.05 was considered statistically significant. Agreement between the TST results (positive, negative or anergy) and QFTGIT results (positive, negative or indeterminate) was assessed using Cohen's Kappa (k) coefficient. K values greater than 0.75, between 0.4 and 0.75, and less than 0.4 were considered excellent, fair and poor agreement, respectively [22].

## Results

### Study participants

A total of 723 study participants, comprising 41.1% (297/723) females and 58.9% (426/723) males, were invited to participate. Of these subjects, 71(9.8%) were excluded owing to common signs/symptoms of active TB or a previous history of TB treatment. Of the remaining 652, 73 did not volunteer to provide blood samples, and blood samples collected from nine individuals were not labelled correctly. Sixty-five (10%) subjects were lost to follow up for TST measurement. Five hundred and eighty-seven (90.0%), 570 (87.4%) and 505 (77.5%) participants had complete data concerning TST, QFTGIT and both tests, respectively. The ages of the 652 study participants ranged from 18 to 70 years, with a median of 30 years. Socio-demographic characteristics and baseline data for the 652 study participants are presented in Table 1.

**Table 1:** Socio-demographic characteristics of study participants

Characteristic	Number (%)
**Gender**:	
Female	262 (40.2)
Male	390 (59.8)
**Age (years)**:	
18-29	269 (41.3)
30-44	241(37.0)
45-60	133 (20.4)
61+	9 (1.4)
**Marital status**:	
Married:	527 (80.8)
Unmarried:	100 (15.3)
Other:	25 (3.83)
**Ethnicity**	
Afar	648 (99.4)
Other	4 (0.6)
**Religion **:	
Muslim:	650 (99.7)
Other:	2 (0.3)
**Occupation **:	
Pastoralist	522 (80.0)
Agro-pastoralist	104 (16.0)
Other	26 (4.0)
**Educational status**	
Illiterate:	574 (88.0)
Literate:	78 (12.0)
**BCG scar present **:	
Yes	113 (17.4)
No	538 (82.6)
**History of contact with TB patient**:	
Yes	44 (6.8)
No	608 (93.3)

### Prevalence of tuberculosis infection

Table 2 demonstrates the prevalence of LTBI assessed by TST and QFTGIT. The prevalence of LTBI was 31.2% (183/587) using TST at a cut-off point ≥ 10 mm, and it was higher in males than females (36.8% vs. 23.5%, X^2 ^= 11.76; P = 0.001). The prevalence of LTBI was not significantly different across age categories (X^2 ^= 3.13; P = 0.372), or between BCG vaccinated and non-vaccinated individuals (37.3% vs. 30.0%; X^2 ^= 2.09; P = 0.148). The prevalence was comparable in individuals who reported a history of close contact with TB patients and those who did not (40.5% vs. 30.6%; X^2 ^= 1.61; P = 0.204). The prevalence of anergy was 48.0% overall (282/587), but the proportion was higher in females than in males (54.3% vs. 43.5%; X^2 ^= 6.59; P = 0.010).

**Table 2:** Prevalence of LTBI by TST and QFTGIT test

Test and Cut-off point	Number (%)
**TST (n = 587)**	
0 mm (anergy)	282 (48.0)
>0 mm & <5 mm	34 (5.8%)
≥ 5 mm & < 10 mm	88 (15.0)
≥ 10 mm	183 (31.2)
**QFTGIT (n = 570)**	
≥ 0.35 IU/ml	363 (63.7)
≥ 0.1 IU/ml	427 (74.9)

**QFTGIT **&**TST (n = 505)**	
≥ 0.35 IU/ml & ≥ 10 mm	151(29.9)
≥ 10 mm	168 (33.3)
≥ 0.35 IU/ml	326 (64.6)

The prevalence of LTBI was 63.7% (363/570) using QFTGIT at the cut-off point recommended by the manufacturer, while it was 74.9% (427/570) at the cut-off point ≥ 0.1 IU/ml of IFN-γ. Six (1.1%) subjects had indeterminate results. The proportion of infection did not significantly differ between genders (male 64.3% vs. female 62.7%; p = 0.908), across age categories (P = 0.363), or between those subjects who reported a history of close contact with TB patients and those who did not (78.1% vs. 62.8%; X^2 ^= 3.19; P = 0.203). The proportion of infection was higher in BCG vaccinated subjects than in non-vaccinated individuals (76.0% vs. 61.2%, X^2 ^= 9.83, P = 0.007). Among the 505 subjects who had TST and QFTGIT test results, 168 (33.3%) tested positive using TST and 326 (64.6%) were positive using QFTGIT (X^2 ^= 70.8; P < 0.001) (Table 2). When subjects with anergy and indeterminate results were excluded from the analysis, the prevalence of LTBI was 61.6% using TST and 83.8% using QFTGIT.

### Agreement between TST and QFTGIT for the diagnosis of LTBI

At the cut-off points **≥ **0.35 IU/ml of IFN-γ for QFTGIT and ≥ 10 mm for TST, 151 (29.9%) subjects tested positive using both assays, whereas 28 (5.5%) subjects were negative in both tests (Table 3). Out of 232 subjects who had skin indurations of 0 mm, 99 (42.7%) tested positive using QFTGIT. The percentage agreement between the overall results (positive, negative and indeterminate/anergy) of the tests (36.04%) was poor (k = 0.098; 95% CI; 0.08 - 0.13)). There was also poor agreement (66.05%) between the results of the tests when only positive and negative results (excluding indeterminate and anergy) were compared (k = 0.195; 95% CI; 0.09 - 0.30). When the cut-off point for positive TST was reduced to ≥ 5 mm of skin indurations, 207 (41.0%) subjects were positive in both tests and six (1.2%) were negative (Table 4). The percentages of agreement between the results (positive, negative and indeterminate/anergy) of the tests were 42.77% (k = 0.136; 95% CI; 0.11- 0.15) and 78.60% (k = 0.058, 95% CI, -0.07 - 0.19) when positive and negative results were considered, respectively.

**Table 3:** Discordant between TST at a cut-off point ≥ 10 mm of skin induration and QFTGIT at a cut-off point ≥ 0.35 IU/m**l **level of IFN-γ

QFTGIT		TST	
	Positive (≥ 10 mm)	Negative (< 10 mm)	Anergy (0 mm)
Positive ( ≥ 0.35 IU/ml)	151	76	99
Negative (< 0.35 IU/ml)	16	28	130
Indeterminate	1	1	3

**Table 4:** Discordant between TST at the cut-off points **≥ **5 mm of skin indurations and QFTGIT at a cut-off point ≥ 0.35 IU/ml level of IFN-γ

QFTGIT	TST		
	Positive (≥ 5 mm)	Negative (<5 mm)	Anergy (0 mm)
Positive ( ≥ 0.35 IU/ml)	207	20	99
Negative (< 0.35 IU/ml)	38	6	130
Indeterminate	2	0	3

**Figure 1:**
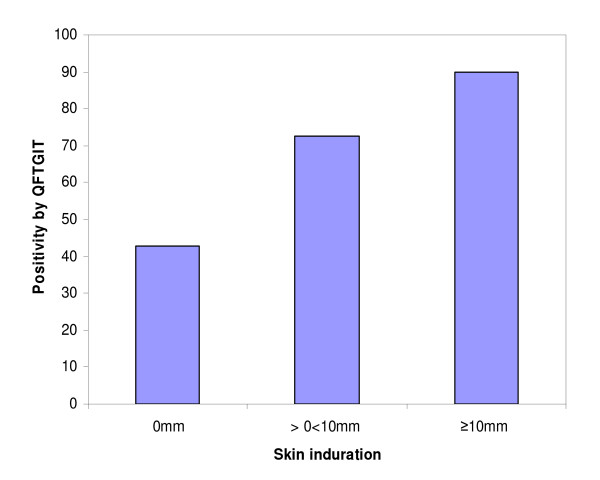
**The trend of proportion of positivity by QFTGIT against the skin test indurations**. Whole blood samples were collected from study participants directly into tubes containing TB-specific antigens, mitogen and nil. The samples were incubated for 24 hours at 37°C, plasma was collected and IFNγ assay was performed using ELISA. The results were interpreted as positive, negative or indeterminate on the basis of the manufacturer's recommended cut-off value (IFN-γ ≥ 0.35 IU/ml) using QFTGIT analysis software developed by the company. Tuberculin skin test (TST) was also performed by administrating 0.1 ml (2T.U/0.1 ml) tuberculin PPD RT23 (Statens Serum Institute, Copenhagen, Denmark) intradermally in the middle of the left forearm. The diameter of the indurations was measured transversely after 48-72 h using a ball-point pen and flexible plastic ruler. The subjects were categorized in to three groups based on the size of skin test induration (0 mm, >0 < 10 mm and ≥ 10 mm) and the positivity by QFTGIT was compared for the three groups.

There was a positive trend between the positivity of QFTGIT and TST (X^2 ^for trend = 96.76; P < 0.001). Figure 1 presents the trend of the proportion of positivity by QFTGIT against the skin test indurations.

### Factors associated with positivity using TST and QFTGIT

Results from logistic regression analysis demonstrated that males were more likely than females to have positivity for LTBI using TST (adjusted OR = 1.8; 95%CI; 1.2 to 2.7; P = 0.004; Table 5). High positivity for QFTGIT was associated with being vaccinated for BCG (adjusted OR = 2.2; 95%CI; 1.2 to 4.1; P = 0.014). Individuals who had skin indurations ≥ 10 mm were 13.6 times more likely to have positive results using QFTGIT than individuals with skin indurations of 0 mm (adjusted OR = 13.6; 95%CI; 7.5 to 24.7; P < 0.001).

**Table 5:** Association of socio-demographic characteristics with TST and QFTGIT positivity

	TST		QFTGIT	
Characteristic	COR (95%, CI)	AOR (95%, CI)	COR (95%, CI)	AOR (95%, CI)

**Gender**:				
Female	Reference	Reference	Reference	Reference
Male	1.9 (1.3 - 2.7)	1.8 (1.2- 2.7)	1.1(0.8 - 1.5)	1.2 (0.8 - 1.7)
**Age (years)**:				
18-29	Reference	Reference	Reference	Reference
30-44	1.1 (0.7- 1.6)	1.1 (0.7 - 1.7)	1.4 (0.9 - 2.0)	0.8 (0.5 - 1.4)
45-60	0.7 (0.4 - 1.2)	0.8 (0.4 - 1.3)	1.7 (1.1- 2.7)	1.3 (0.7 - 2.4)
61+	1.7(0.5 - 6.5)	1.8 (0.4 - 7.1)	2.4 (0.5 - 11.7)	1.4 (0.2 -8.2)
**Marital status**:				
Married:	Reference	Reference	Reference	Reference
Unmarried:	1.3 (0.8 - 2.0)	0.9 (0.5 - 1.6)	0.5 (0.3 - 0.8)	0.5 (0.2 - 0.9)
Other:	0.5 (0.2- 1.4)	0.5 (0.2 - 1.6)	1.0 (0.4- 2.8)	1.6 (0.5 - 4.9)
**Occupation **:				
Pastoralist	Reference	Reference	Reference	Reference
Agro-pastoralist	1.2 (0.7 - 1.9)	0.9 (0.6 - 1.5)	1.4 (0.9 - 2.3)	1.5 (0.9 - 2.6)
Other	2.1 (0.8 - 5.3)	1.4 (0.5 - 3.8)	1.3 (0.5- 3.3)	1.7 (0.6 - 4.7)
**Educational status**				
Illiterate:	Reference	Reference	Reference	Reference
Literate:	2.2 (1.3 - 3.6)	1.7 (0.9 - 3.0)	1.0 (0.6 -1.7	1.1 (0.6 - 1.9)
**BCG scar present**				
No	Reference	Reference	Reference	Reference
Yes	1.4 (0.9 - 2.2)	1.4 (0.9 - 2.2)	2.2 (1.3 - 3.6)	2.2 (1.2 -4.1)
**Contact with TB pts**				
No	Reference	Reference	Reference	Reference
Yes	1.6 (0.8 - 3.1)	1.6 (0.8 - 3.3)	2.0 (0.9 - 4.7)	2.0 (0.8 - 4.9)
**TST**				
0 mm			Reference	Reference
> 0 < 10 mm			3.5 (2.1- 5.8)	3.5 (2.1- 5.8)
≥ 10 mm			12.1(6.8 - 21.6)	13.6 (7.5- 24.7)

## Discussion

In this study, the prevalence of LTBI in the pastoral community of Afar, one of the regions in Ethiopia with a high TB notification rate [1], was assessed. The TST results revealed a prevalence of 31.2% for LTBI among study participants who had complete TST results. This is comparable with the estimation that one-third of the world's population is infected with *Mycobacterium tuberculosis *(Mtb) [3], and with the prevalence of LTBI reported in India using TST [21]. However, it is lower than the prevalence of LTBI reported in HIV-negative adult factory workers from Ethiopia [31] and from adult study participants in South Africa [32]. The discrepancy between the results of the present study and those of previous studies carried out in Ethiopia could be explained by several factors including the study setting, the BCG status or immune profile of the study participants, and other socio-demographic factors [23,31].

The QFTGIT test results demonstrated a high prevalence of LTBI compared with TST, similar to the findings of a study concerning TB prevalence among healthy adults in a high endemic area of India [21]. A study conducted in a cohort of health care workers in India demonstrated a similar prevalence of LTBI using QFTGIT (40.1%) and TST (41.4%) [17]. However, a previous study that compared the diagnostic efficacy of the old QuantiFERON-TB to that of TST in study participants from Ethiopia and Baltimore indicated that TST would be more effective for diagnosing LTBI in Ethiopian individuals [33]. The results of a study conducted in South Africa also suggested that TST was more effective in identifying LTBI than three generations of a whole blood IFN-γ assay in countries with a high TB burden [22]. The discrepancy between the present study and previous studies could be explained by several factors including differences in the study setting, socio-demographic factors of the study participants, BCG status of individuals and the type of IGRAs test used. For instance, in the study involving Ethiopian and American individuals [33], the authors used PPD tuberculin (Tubersol; Pasteur Merieux Connaught Laboratories) and defined skin indurations of ≥ 5 mm as a positive reaction in Ethiopian subjects, while the current study defined skin indurations of ≥ 10 mm as positive for LTBI [29].

There is evidence that IGRAs are more sensitive than TST for detecting LTBI in various groups of immune-compromised individuals [10,34,35]. In the present study, among the 232 study subjects who had no measurable skin indurations (0 mm), 99 (42.7%) tested positive using QFTGIT. Similarly, among 105 subjects who had skin indurations < 10 mm, 72.4% tested positive using QFTGIT. This indicates that the use of QFTGIT for the epidemiological study of LTBI is feasible, even in populations unreactive to TST owing to diminishing immune responses [10,34]. However, 16 subjects who had skin indurations ≥ 10 mm (of whom only three had a BCG scar) were negative using QFTGIT. This finding is similar to a study carried out in South Africa, where a considerable proportion of individuals who had skin indurations ≥ 15 mm tested negative using QuantiFERON tests [22]. This was likely to be due to false positive results using TST, as infection with non-tuberculosis mycobacteria would cause an individual to test positive [5]. It is reasonable to suppose that false negative results using QFTGIT could occur as the present cut-off value recommended by the manufacturer is thought to be too high, as suggested by this study and others [14,36]. The possibility of false positive results due to non-tuberculosis mycobacterial infections is greater using TST than IGRAs, but it is possible that the high positive results observed using QFTGIT in the present study could be due, in part, to cross-reactivity of specific antigens with non-tuberculosis mycobacterial infections [37,38]. Therefore, further studies are required to investigate if there are factors that could contribute to the high positive results obtained in the present study area using QFTGIT, other than infection with Mtb complex.

There was poor agreement between the TST and QFTGIT results. In the present study setting, where TB is endemic [1,25], repeated exposure to Mtb and the presence of more persistent memory T cells than effector cells would be expected, and therefore an increased response to PPD [28,39,40]. However, the dynamics of the immune response to Mtb infection are still unclear [15]. Nevertheless, in the present study, the main cause of disagreement was due to a high proportion of positive results using QFTGIT and a high proportion of negative results using TST, similar to the findings of a previous study [21], but different to those reported by Kang et al. [41]. The high prevalence of anergy using the TST and the low prevalence of indeterminate results using QFTGIT could contribute to the disagreement between the two tests. Anergy to TST could occur because of technical problems, malnutrition, other infectious agents such as HIV/AIDS and parasitic infections, or the absence of tuberculosis infection. However, the higher proportion of anergy to TST in female study participants and the lower proportion of positive results using QFTGIT in anergic individuals imply that anergy in the present study is likely to have arisen from factors other than technical problems related to TST. However, the lower proportion of positivity observed in anergic individuals using QFTGIT indicates that skin indurations of 0 mm in most individuals are probably due to the absence of infection with Mtb complex rather than anergy. Therefore, further studies are required to elucidate the role(s) of possible factors such as malnutrition, HIV/AIDS and other infectious agents associated with anergy or false negative results using TST in the present study area.

Despite the poor agreement between the two tests and the greater effectiveness of QFTGIT than TST in detecting LTBI, there was a trend towards an increased proportion of positivity using QFTGIT with increasing TST reactivity (Figure 1); this corroborates the findings of a study carried out by Adetifa et al. [42]. This trend between the two tests may suggest that both the specific antigens and PPD stimulate the same T cell population and measure the same response, contradicting evidence that IGRAs measure the response of effector T cells and that the TST measures memory T-cell responses [28,39,40]. Furthermore, like TST, IGRAs are susceptible to factors affecting host immune responses, although the degree of susceptibility differs between the two tests.

In this study, the prevalence of LTBI was higher in men than women, as estimated using TST. The finding corroborates the result of a study carried out in South Africa [32], but the low prevalence of LTBI in women could be due to the high prevalence of anergy observed in female study participants. However, the prevalence was not altered by gender or by age groups when estimated using the QFTGIT test. This could be additional evidence that anergy contributes to the low prevalence of LTBI in women. Studies have demonstrated a high risk of Mtb infection among individuals in close contact with infectious TB cases [43,44]. However, in the present study, the positivity of TST or QFTGIT was not affected by a previous history of close contact with TB patients, and this is in agreement with the findings of a study carried out in South Africa [22]. This could be explained by the high endemic nature of TB, hampering the association between the prevalence of LTBI and TB case contact. In other words, in areas highly endemic for TB, individuals can acquire infection not only through close contact with TB patients, but also from the community. Previous BCG vaccination was not associated with positive TST. This finding is consistent with the results from study carried out in other highly endemic country [22], and differs from the results of studies carried out in countries with low TB prevalence [45,46]. The lack of an effect of BCG on the results obtained using TST implies that TST can be of use for the screening of LTBI in highly endemic areas, particularly in adult individuals, when the effect of the BCG has waned [47]. However, the high positivity using QFTGIT was associated with the presence of a BCG scar. This finding is consistent with the findings of Tsiouris et al. [48], who evaluated the use of IGRAs for the diagnosis of TB using QFTGIT in an area highly endemic for TB. These findings warrant further investigation as IGRAs are not supposed to be confounded by prior BCG vaccination.

## Conclusions

QFTGIT demonstrated a higher prevalence of LTBI than TST in the present study area. Unlike TST, the QFTGIT test results were not affected by gender or the age of study participants. This suggests that QFTGIT could be used for the epidemiological study of LTBI at a community level, even in a population unreactive to TST, although there is currently no agreed gold standard for the diagnosis of LTBI. Nevertheless, further studies in adults and children are required to assess the effect of factors such as malnutrition, non-tuberculosis mycobacterial infections, HIV and parasitic infections on the performance of the test. The high prevalence of LTBI observed in the present study area could have implications for the rising prevalence of active TB in the area, particularly when favourable conditions including malnutrition and HIV/AIDS coexist. Therefore, any possible intervention that minimizes the risk of progression of LTBI to active TB is important and its uses should be thoroughly investigated.

## Competing interests

The authors declare that they have no competing interests.

The study was financially supported by Norwegian Programme for Development, Research and Education, NUFU (NUFU PRO-2007/10198).

## Authors' contributions

ML designed the study, participated in data collection, analysis and drafted the manuscript. GA, participated in study design, data collection, analysis and write-up. GM participated in study design, data collection and write-up. GMD, participated in study design, data analysis/interpretation and write-up. GB involved in study design and write-up of manuscript. FA involved in study design, data analysis and write-up of the manuscript and critically revised the manuscript. All authors read and approved the final manuscript. ML is the guarantor of the paper.

## Pre-publication history

The pre-publication history for this paper can be accessed here:

http://www.biomedcentral.com/1471-2334/11/89/prepub
